# Electronic Health Program to Empower Patients in Returning to Normal Activities After Colorectal Surgical Procedures: Mixed-Methods Process Evaluation Alongside a Randomized Controlled Trial

**DOI:** 10.2196/10674

**Published:** 2019-01-29

**Authors:** Chantal M den Bakker, Judith AF Huirne, Frederieke G Schaafsma, Charlotte de Geus, Hendrik J Bonjer, Johannes R Anema

**Affiliations:** 1 Department of Occupational and Public Health Amsterdam Public Health Research Institute VU University Medical Center Amsterdam Netherlands; 2 Department of Surgery VU University Medical Center Amsterdam Netherlands; 3 Department of Gynecology VU University Medical Center Amsterdam Netherlands

**Keywords:** return to normal activities, return to work, patient reported outcome measures, colectomy, process evaluation

## Abstract

**Background:**

Long-term recovery takes longer than expected despite improved surgical techniques and Enhanced Recovery After Surgery programs. An electronic health (eHealth) care program (“ikherstel”) was developed to partially substitute perioperative care for patients undergoing colorectal surgical procedures. Successfully tested eHealth programs are not always implemented in usual care, and it is, therefore, important to evaluate the process to optimize future implementation.

**Objective:**

The aim of this study was to evaluate whether the eHealth intervention was executed as planned.

**Methods:**

A mixed-methods process evaluation was carried out alongside a multicenter randomized controlled trial (RCT). This evaluation was performed using the Linnan and Steckler framework for the quantitative part of this study, measuring the components reach, dose delivered, dose received, fidelity, and participants’ attitudes. Total implementation scores were calculated using the averaging approach, in which the sum of all data points is divided by the number of data points and the total adherence to the protocol is measured. For the qualitative part, the Unified Theory of Acceptance and Use of Technology framework was used. The quantitative data were based on participants’ questionnaires, a logistic database, a weblog, and participants’ medical files and were obtained by performing semistructured interviews with participants of the RCT.

**Results:**

A total of 151 participants of 340 eligible patients were included in the RCT, of which 73 participants were allocated to the intervention group. On the basis of the quantitative process data, total implementation scores for the website, mobile app, electronic consult, and activity tracker were 64%, 63%, 44%, and 67%, respectively. Participants in the qualitative part experienced the program as supportive and provided guidance on their recovery process after colorectal surgery. Most frequently mentioned barriers were the limited interaction with and feedback from health care professionals and the lack of tailoring of the convalescence plan in case of a different course of recovery.

**Conclusions:**

The intervention needs more interaction with and feedback from health care professionals and needs more tailored guidance in case of different recovery or treatment courses. To ensure a successful implementation of the program in daily practice, some adjustments are required to optimize the program in a blended care form.

**Trial Registration:**

Netherlands Trial Registry NTR5686; http://www.trialregister.nl/trialreg/admin/rctview.asp?TC= 5686 (Archieved by WebCite at http://www.webcitation.org/75LrJaHrr)

## Introduction

### Background

In-hospital stay after colorectal surgical procedures has shortened enormously attributable to improved surgical techniques and Enhanced Recovery After Surgery programs, but further recovery at home still takes longer than expected by health care professionals [1-4]. Electronic health (eHealth) can be a suitable tool to optimize perioperative care including at-home recovery by providing tailored information, increasing patients’ self-management, and by delivering interactive communication features. Patients can become their own empowered and motivated health managers [5-8].

To partially substitute guiding and monitoring of long-term recovery including resumption of normal activities and work of colorectal patients, an eHealth intervention called “ikherstel” or “I recover” was developed using the intervention mapping protocol. This innovative eHealth program consists of, among others, a website, a mobile app, an activity tracker, and the possibility of an electronic consult (eConsult). The program will be evaluated in a multicenter single blinded randomized controlled trial (RCT) [9].

Many eHealth care programs are developed and tested in health care; however, successful eHealth programs will not automatically be implemented in usual daily care in all cases. An integral part of evaluating successful eHealth interventions is measuring the adherence to the intervention protocol, as this will play an important role in interpreting the results regarding the effectivity and it might improve further implementation [10-12]. Measuring adherence and compliance to perioperative care processes is a fundamental aspect in improving the quality of surgical care [13]. It is also desirable for optimal implementation to evaluate how well the intervention was appreciated by participants, and a process evaluation can contribute to this [14]. The quantitative data in a mixed-methods approach contribute to understanding why a (complex) intervention has its intended impact, if any, and in which domain this went as planned or not [15]. In addition, by using qualitative data, patients’ experiences including barriers and facilitators may be reviewed in more detail to adjust the eHealth care program for future implementation.

### Objectives

The aim of this study was to evaluate whether the recovery-orientated eHealth intervention was executed as planned. This can help to conduct per protocol analyses, to assist with interpreting the future trial outcomes, and to determine important factors for program scale up in this specific cancer-dominated study population.

## Methods

### Trial Design

A mixed-methods process evaluation of quantitative data obtained from participants’ questionnaires, a logistic database, a weblog, and participants’ medical files and a qualitative analysis of semiconstructed interviews were conducted. This evaluation was carried out alongside a multicenter, single-blinded RCT in 10 teaching hospitals in the Netherlands and is reported in accordance with the Consolidated Standards of Reporting Trials of Electronic and Mobile Health Applications and onLine TeleHealth (CONSORT-EHEALTH) [16]. The intervention development including trial protocol has been published previously [17]. This RCT was approved by the Medical Ethics Committee of the VU University medical center under registration number 2014.301. Under registration number NTR5686, this study was also registered at the Netherlands Trial Registry.

### Participants

Patients aged between 18 and 75 years who were scheduled for a laparoscopic or abdominal colorectal resection or hysterectomy were eligible to participate in the RCT. This process evaluation describes all participants who underwent a colorectal resection with malignant and benign indications. Exclusion criteria were (1) surgery without a curative intention, (2) concomitant surgical procedures, (3) not able to use the internet, (4) unable to understand Dutch questionnaires, and (5) receiving neoadjuvant treatment. For the interviews, purposeful sampling was used including participants with both a positive and a negative rating of the program.

### Intervention

Participants were randomized and allocated to the intervention or control group in a 1:1 ratio by a researcher who was independent from the recruitment, data collection process, or analyses. Study participants were blinded to the allocation. Participants in the control group received usual care and access to a placebo website, which contained a patient information brochure about the surgical procedure. Participants in the intervention group received access to an innovative eHealth care program (called “ikherstel”-intervention or “I recover”-intervention). This program consisted, among others, of a website, a mobile phone app, an activity tracker, and the possibility to ask questions to health care professionals of their own hospital via an eConsult. All included functionalities can be found in the intervention mapping study [17].

### Data Collection

Participants filled out an adherence and satisfaction questionnaire 3 months after the surgical procedure. In addition, a logistic database, a weblog, and participants’ medical files were used to collect quantitative data. Semistructured interviews were conducted in October and November 2017, which was approximately 1 year after inclusion of the first patient in the RCT who participated in the qualitative part of this study. The topics were created to gather information about patients’ barriers and facilitators for use of the intervention. In the preparation phase, topics and questions were created based on literature, the theoretical framework, and discussion with the project team. All interviews were recorded with a voice recorder. Informed consent was obtained initially when patients participated in the RCT.

### Theoretical Frameworks and Process Outcomes

**Figure 1:**
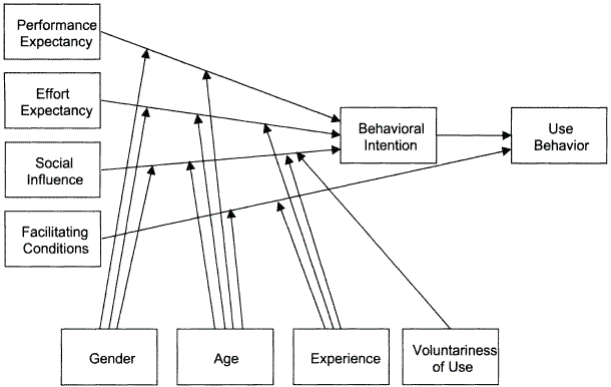
The Unified Theory of Acceptance and Use of Technology framework (UTAUT) for the qualitative part of the study.

The Linnan and Steckler model was used for the quantitative part of the process evaluation [18]. This is a commonly used model and has the potential to systematically evaluate the process of implementation. The adherence to the intervention is described in 5 terms: (1) the proportion of intended target audience that participated in the study—reach, (2) the number of intended units of each component that was delivered to the intervention group—dose delivered, (3) the number of participants from the intervention group that actively engaged the delivered components of the intervention—dose received, (4) the extent of the intervention that was delivered as planned—fidelity, and (5) participants’ satisfaction and usage barriers of the intervention—participants’ attitudes. The components were assessed for each function separately, except the component participants’ attitudes for eConsult. Operationalization per component per process outcome is further explained in the Results section. Total implementation scores were calculated by using the averaging approach, in which the sum of all data points is divided by the number of data points and the total adherence to the protocol is measured. The Unified Theory of Acceptance and Use of Technology framework (UTAUT) was used for the qualitative part of this evaluation [19]. This framework integrates fragmented theories and research on individual acceptance of information technology into 1 model with 4 core constructs to help gain insight into (1) the degree patients’ believe that using the “ikherstel” program will help in their recovery—performance expectancy, (2) the ease of use patients experienced when using the “ikherstel” program—effort expectancy, (3) the degree to which patients perceive that it is important others believe that he or she should use the “ikherstel” program—social influence, and (4) other external factors that facilitate or inhibit the use of the “ikherstel” program—facilitating conditions. These constructs measure the impact of behavioral intention and use behavior. These can play a role as determinants of user acceptance. Overall, 4 moderators (gender, age, experience, and voluntariness of use) were incorporated into this framework. However, it was decided to not measure these moderators, given the qualitative nature of this part of the study. A detailed description of this framework is provided in Figure 1.

### Data Analysis

IBM SPSS Statistics version 22.0 was used for analyzing the quantitative data. These data were analyzed using descriptive statistics such as frequencies, means, and SDs. The semistructured interviews for the qualitative part were recorded and transcribed verbatim. Each participant was allocated a study number, and all names were removed from the transcripts to ensure an anonymous analysis. After transcription, the researchers first familiarized themselves with the data and read the transcripts thoroughly. Subsequently, the verbatim transcripts were analyzed comprising open, axial, and selective coding.

Overall, 25% of the transcripts were coded and analyzed by a second independent researcher to reduce investigator bias and improve validity and inter-rater reliability. Furthermore, the level of data saturation was systematically studied. The frequency of quotes within each theme and their distribution across the interviews were explored, based on a data saturation approach as described by Guest et al [20]. Cited quotes were translated directly from Dutch and were added to illustrate the themes. The qualitative data analysis ATLAS.ti software (version 7.0, Scientific Software Development) was used.

## Results

### Quantitative Part

#### Reach

**Figure 2:**
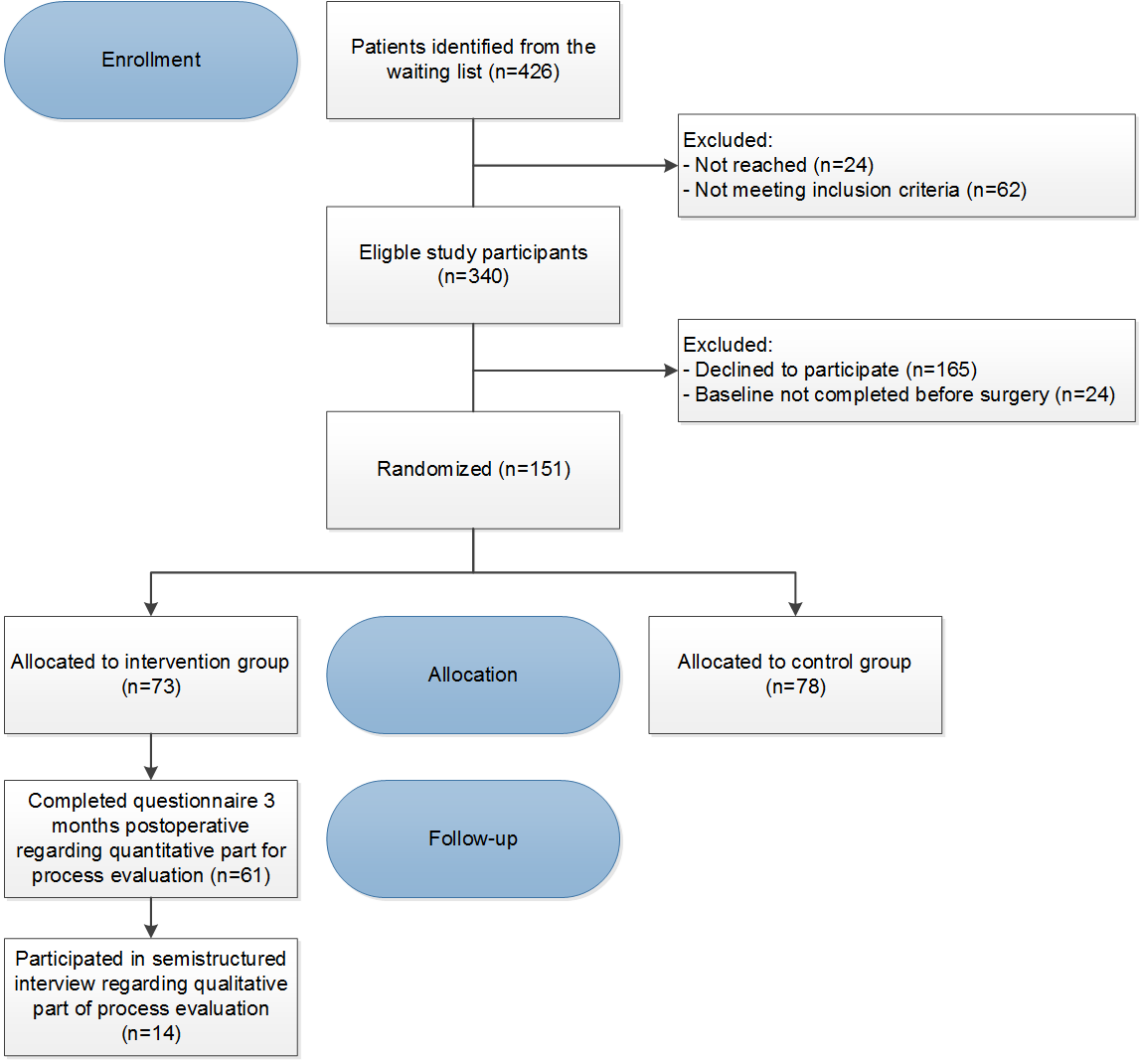
Flow diagram of the inclusion process.

During February 2016 and September 2017, a total of 426 patients scheduled for a colorectal resection were invited to participate in the RCT. Of these patients, 62 patients were ultimately not eligible to participate and 24 patients did not reach on time. Of the 340 suitable patients, 151 patients agreed to participate in the trial (44.4%) and gave informed consent. A total of 73 colorectal participants were allocated to the intervention group and received the recovery-orientated eHealth program. The flow diagram of the inclusion process is presented in Figure 2. Moreover, 69% (50/73) of the patients were male and their mean age was 62.6 years, and 56 patients (77%, 56/73) had a colon or rectal carcinoma. All baseline characteristics are presented in Table 1. A total of 61 participants completed the quantitative evaluation questionnaire 3 months after surgery, which is considered a representative sample with respect to all baseline characteristics. All other scores per domain per intervention functionality and how this has been operationalized in this study are presented in Table 2.

**Table 1:** Baseline characteristics (N=73).

Variable		Intervention group
**Gender,** **n (%)**		
	Female	23 (32)
	Male	50 (69)
Age, mean (SD)		62.6 (7.8)
**Nationality,** **n (%)**		
	Dutch	73 (100)
	Other	0 (0)
**Education level,** **n (%)**		
	Low	20 (27)
	Medium	31 (43)
	High	22 (30)
**Work status,** **n (%)**		
	Employed	36 (49)
	Not employed	37 (51)
**Type of surgery,** **n (%)**		
	Abdominal procedure	4 (6)
	Laparoscopic procedure	69 (95)
**Indication,** **n (%)**		
	Benign	17 (23)
	Malignant	56 (77)

**Table 2:** Results of the quantitative part of the study according to Linnan and Steckler. 151 (44.4%) patients who met the inclusion criteria signed informed consent and were randomized to the intervention or control group.

Reach	Website	App	Electronic consult	Activity tracker
Dose delivered	73 (100%, 73/73) patients who received an account/all patients of the intervention group	59 (81%, 59/73) patients who received an account for the app/all patients of the intervention group	73 (100%, 73/73) patients who received an account/all patients of the intervention group	57 (78%, 57/73) patients who received an activity tracker/all patients of the intervention group
Data collection	Logistic database	Logistic database	Logistic database	Logistic database
Dose received	60 (82%, 60/73) patients who made a convalescence plan/patients who received an account	39 (66%, 39/59) patients who installed the app/patients who received an account and completed the questionnaire	4 (6%, 4/73) patients who asked a question about the Web portal/patients who received an account for the Web portal	39 (68%, 39/57) patients who connected the activity tracker to their phone/all patients that received an activity tracker
Data collection	Weblog	Weblog	Weblog	Weblog
Fidelity	17 (28%, 17/60) patients who used the website/patients who made a convalescence plan	24 (62%, 24/39) patients who used the app/patients who installed the app and completed the questionnaire	1 (25%, 1/4) questions that are answered/questions that are asked	30 (77%, 30/39) patients that used the activity tracker/all patients that connected an activity tracker
Data collection	Questionnaire	Questionnaire	Weblog	Weblog+Questionnaire
Participants’ attitude	Mean score 7.1 (1-9)	Mean score 7.5 (1-10)	N/A^a^	Mean score 7.1 (1-10)
Data collection	Questionnaire	Questionnaire	N/A	Questionnaire
Implementation score	64% (the sum of all data points/by the number of data points)	63% (the sum of all data points/by the number of data points)	44% (the sum of all data points/by the number of data points)	67% (the sum of all data points/by the number of data points)

^a^N/A: not applicable.

#### Dose Delivered

All participants (100%) received an account for the intervention website and thereby the ability to use the eConsult functionality (100%). Overall, 59 participants had a suitable mobile phone for the mobile app (81%, 59/73), and 57 participants had a suitable smartphone to use the activity tracker (78%, 57/73).

#### Dose Received

Overall, 60 participants made a convalescence plan on the intervention website (82%, 60/73). A total of 39 participants downloaded the app (66%, 39/59) and connected the activity tracker with their smartphone (68%, 39/57). Moreover, 4 participants used the possibility of an eConsult (6%, 4/73).

#### Fidelity

Overall, 17 participants used the website more than once (28%). Of the 39 participants who had downloaded the app, 24 participants used it (62%). In addition, 13 participants used the app in combination with the website and the remaining 11 participants only used the app. Overall, 4 participants only used the website. Moreover, 1 out of the 4 questions asked via the eConsult option was answered by a health care professional (25%). Of the 39 participants that connected the activity tracker, 30 participants actually used it (77%).

#### Participants’ Attitude

Mean ratings for the website, app, and activity tracker were 7.1 (range 1-9), 7.5 (range 1-10), and 7.1 (range 1-10), respectively.

#### Implementation Scores

Total implementation scores for the website, the mobile app, the eConsult, and the activity tracker are 64%, 63%, 44%, and 67%, respectively.

### Qualitative Part

A total of 14 semistructured interviews were conducted during November and December 2017. The overall mean age at the time of the interview was 62 years (range 45-76 years); 4 participants were female and 10 were male. The characteristics of these 14 participants are shown in Multimedia Appendix 1. Most important findings are discussed below per construct regarding the UTAUT model. An overview of all findings is presented in Table 3.

#### Performance Expectancy

The key themes (1) guidance and support, (2) information provision, (3) communication, and (4) functionality were identified out of the analysis.

##### Guidance and Support

Participants were positive about the “ikherstel” program. They found it supporting and guiding in their recovery process after colorectal surgery. Participants stated that the program was a good guideline and a good way of monitoring their progress. This knowledge gave the participants a feeling of reassurance and security about their recovery:

Security. A feeling of confidence, whether I am going in the right direction. Naturally, you do not continuously call a doctor or the hospital to check whether it is going okay, or I feel this or I feel that. Because of this app you know, you have to meet these requirements, so it is all right.

Participant 3, male, 54 years

The participants also perceived the program as effective in progressing their recovery, whereby participants felt that the “ikherstel” program had achieved its goal. Furthermore, the participants were positive about the activity tracker or the concept of an activity tracker. Participants agreed with the notion that using the activity tracker motivated them to be active and that it was a good way to reflect on their level of activity. It provided a goal to work toward and the steps they had to take to get to this goal. Participants stated that the difference between what they thought their level of activity was and what the activity tracker showed could either motivate them to be more active the following day or positively surprised them and generated a positive and satisfied feeling:

But that does stimulate you at the end of the day, to see where I am and “oh tomorrow I have to do a bit more.”

Participant 12, female, 65 years

When participants did not fully adhere to the personalized recovery plan, this was generally because participants recovered faster than the recovery plan advised to them. Therefore, the provided recommendations were sometimes reviewed as too conservative. As a result, these participants would follow their own instinct and resumed activities when their body felt ready for it.

##### Information Provision

Participants stated that the information provided on the website was insightful, useful, and all-encompassing. However, not all patients felt the need to read the extra information on the website as they already received sufficient information and guidance from their treating health care professionals. Participants mentioned 3 advantages of the provided information on the website: (1) the information was always available; (2) the information came from a reputable and, therefore, trustworthy source; and (3) the information was more elaborate than they had received in the hospital. These advantages were considered relevant as participants mentioned that it was easy to forget what was told in the hospital and they did not always have time to discuss everything in the hospital:

It is also information that you otherwise do not get. Visiting the doctor is always pretty quick of course. The time I was in the hospital, yes you can of course ask all the questions to the nurse, but this was just a little bit more. And you can also look it up again. It was nice.

Participant 12, female, 65 years

**Table 3:** Key findings for each of the 4 constructs of the Unified Theory of Acceptance and Use of Technology model.

Construct	Topic	Elaboration
Performance expectancy	Supporting and guiding	The “ikherstel” program supported and guided the recovery process after surgery. It provides good opportunities to monitor your own recovery process as well as a goal to work toward.
	Activity tracker motivates	The activity tracker motivated to be physically active.
	Deviated from recovery plan	Some participants did not adhere to the personalized recovery plan. Reasons for deviation included advanced recovery before the recovery plan resulting in resumed activities when the participants’ body felt ready for it.
	Psychological aspects should be included	Participants stated there is too much focus on physical recovery, whereas psychological aspects were not taken into account. According to the participants, psychological well-being and a positive attitude to cope with the emotional burden of cancer diagnosis were an integral part of recovery.
	More personalized	Participants would have preferred it to be more personalized, including more focus on individual aspects and needs of the patient and inclusion of social conditions that influence the recovery process.
	Useful and insightful	The provided information on the website was found to be useful and all-encompassing. Some participants indicated that they did not need the information or that they did not read it. Some participants also desired extra information on diet and prevention and more extensive information on symptoms and complications.
	Information on the websites provides advantages	It was easy to forget what was said in the hospital and the website provided a good backup. Participants only had a short amount of time to ask questions in the hospital, and therefore, it was good to have the website that provided additional information. The information on the website was readily available. Participants felt the information was trustworthy as it came from a reputable information source.
	Need for more feedback and interaction	There was a need for more personal interaction and feedback on progress of the recovery process. There was a one-way information stream from the patient to the “ikherstel” program; this has to become a two-way information stream.
	More involvement of hospital or doctor	Participants desired more involvement and feedback from the hospital or treating doctor.
	Functionalities have to work correctly	The activity tracker experienced problems with connection and did not function properly in some cases. After consultation via electronic consult, no answer was given, whereas this should have been given within 2 days.
Effort expectancy	Easy to use	The “ikherstel” program was found to be easy to use and it costed no effort.
	More support in setting up the program	Some participants stated they would have appreciated more support during the program’s start-up phase.
Social influence	Would recommend the program	The majority of participants would recommend the “ikherstel” program to their family and friends.
	Social influence from health professional	Family and friends had little or no social influence. There was more social influence from the hospital and doctor.
Facilitating and inhibiting conditions	Inflexible in case of alternative disease course	An inhibiting factor was the inflexibility of the personalized recovery plan in case of a deviant recovery course, which can occur in case of complications.
	Insufficient for prolonged disease course (chemotherapy)	The “ikherstel” program was insufficient for patients who received chemotherapy due to the prolonged disease course and the additional needs (eg, additional information on helpful methods to cope with chemotherapy and prolonged use activity tracker).
	Level of information provision from hospital	The information provision from the hospital influenced the need for the “ikherstel” program. This could be either a negative influence in case of adequate information provision or positive in case of a lack of information provision.
	Positive attitude	A positive attitude facilitated the recovery process and therefore also the use of the “ikherstel” program.
	Physical fitness before surgery	Physical fitness before surgery influenced the postoperative recovery process.

##### Communication

Despite the advantages of the “ikherstel” program, participants stated that there is still much to be improved in relation to communication. Currently, there is mostly a one-way information stream from the user to the “ikherstel” program with the exception of eConsult. Participants would also appreciate this interaction in other functionalities of the intervention. In the current situation, the user provided information about their recovery progress to the program. However, no interactive feedback was provided on whether the patient was on track, and no follow-up questions were asked in case a patient was not able to keep up. Furthermore, patients felt the need to hear how they were doing. The graphically displayed progress on the website and in the mobile app was not enough; they had a need to hear it from the involved health care professional, which gave them more reassurance. In the current set-up, the doctor was not engaged and up-to-date in the recovery process of the participant at home. Participants valued the opinion of their doctor as very important, and therefore, it is important that the “ikherstel” program is supported by the hospital. They mentioned that this might also prevent conflicting messages in recovery advice:

And also some feedback from the hospital, from the treating doctor, the surgeon's assistant that he performs a few calls to check and ask how everything goes and helps a little. I think that, that is the solution is to achieve huge benefits.

Participant 5, male, 74 years

##### Functionality

Participants recognized the added value of the various functionalities. Therefore, it was important that all the functionalities work properly, including the activity tracker and eConsult. Some participants had problems with the activity tracker that was provided with the “ikherstel” program. Even though the activity tracker did not always work properly, participants recognized the added benefit of the concept of an activity tracker and the idea behind it. Therefore, as an alternative for the malfunctioning activity tracker, some participants replaced the provided activity tracker with a built-in step counter on their smartphone or they downloaded an independent activity-tracking app on their mobile phone. This gave them the same opportunity of tracking their activity without the problems that came with the activity tracker that was provided by the “ikherstel” program:

The activity tracker was understandable but again that whole synchronization just did not work well. Yes, I did not understand that. I thought that was unfortunate. So I started using my iPhone at a given time. Because that also shows the amount of steps per day.

Participant 10, male, 64 years

Participants suggested to include a functionality to check their psychological well-being instead of just focusing on physical recovery. Participants stated that psychological well-being and a positive attitude after cancer surgery influenced recovery and should, therefore, be an integral part of the recovery process. Including a functionality that investigates psychological well-being in the “ikherstel” program would be of added value according to participants.

Participants appreciated the fact that they always had the app at hand and could easily and frequently check their recovery progress during the day. In the beginning, participants used the website to make the personalized recovery plan and read the information on the website, and then, they switched to the app. Participants, therefore, recognized the app as an extra benefit of the “ikherstel” program.

#### Effort Expectancy

Participants found the “ikherstel” program easy to use, practical, and easy to fit into their daily life with the exception of the activity tracker. Participants stated that it does not cost any effort to use the “ikherstel” program. However, it was mentioned that in the beginning it did cost effort to set up the “ikherstel” program and to find out how it works. Participants suggested that it would be useful if more help would be available for setting up the “ikherstel” program:

Practical. Easy. Yes, you could just fit it into your daily life.

Participant 12, female, 65 years

#### Social Influence

There was little or no social influence of family and friends on participants in using the “ikherstel” program. Participants had approached the intervention very individualistically. When participants informed family and friends about the “ikherstel” program, they were either positive about the fact that the patient participated or they had no opinion about it. Social influence from health care professionals is also quite relevant in this construct. More involvement of health care professionals with the “ikherstel” program is suggested so that patients feel engagement and support from the doctor and hospital:

I've seen it has been established and I got the feeling that it was fixed. So it would be nice if someone looks at the recovery monitor and sees that a patient stays behind in certain areas or goes very quickly in certain areas and that the recovery plan can be adjusted accordingly. So that it really becomes interactive.

Participant 14, male, 48 years

#### Facilitating and Inhibiting Conditions

##### Alternative Course of Disease

A major inhibiting condition for future usage of the “ikherstel” program is the inflexibility of the personalized recovery plan in case of an alternative course of disease. This can happen, for example, in case of complications or additional surgical procedures (ie, removal of a stoma at a later stage). In case the recovery process of the participant took longer, there was currently no option to adjust the personalized recovery plan. Due to this inflexibility, the “ikherstel” program was graded as insufficient by these participants. Participants stated there should be an option to revise the recovery plan in case of an alternative course of disease to their new situation:

Some things happened in between, I had an extra hospital admission, as a result I found that “ikherstel” did not fit well with the situation I was in.

Participant 10, male, 64 years

##### Chemotherapy

The “ikherstel” program was also rated insufficient by participants who received chemotherapy in addition to their surgery. For these participants, the surgical procedure was considered more as a side issue, whereas the “ikherstel” program was targeted mainly at recovering from colorectal surgery. Participants who received chemotherapy had a need for additional supportive care after recovery from surgery. They also had a desire for prolonged use of the activity tracker. Participants used the tracker in the first 8 weeks after surgery, and this motivated them enormously. After 6 to 8 weeks, chemotherapy started, but after 8 weeks, the activity tracker had to be sent back to the researcher:

Now it was in my opinion much more focused on ehh well you've had an operation. How do you recover from that operation and that kind of thing. But not afterwards of, okay, ehh now there follows chemotherapy, what does that mean and what can we offer in the program to help people. It is certainly helpful.

Participant 6, male, 64 years

## Discussion

### Principal Findings

This process evaluation used both a quantitative and qualitative approach to evaluate the implementation process of a recovery-orientated eHealth intervention. In the quantitative part, average implementation scores per functionality of approximately 60% were reached. A mean score of 7.1 out of 10 for the website, 7.5 for the mobile app, and 7.1 for the activity tracker was given by participants. Barriers and facilitators for use were identified in the qualitative part of this evaluation. The program was experienced by participants as supportive and provided guidance on their recovery progress. Participants had a positive view about the concept and the ease of use of the “ikherstel” program. It was reported that not all functionalities of the program worked properly, and for further sustainable implementation, this needs to be optimized. Interaction with and feedback from health care professionals was a preferred feature by participants, which is currently lacking. In case participants had a different course of recovery, a complication, or received adjuvant chemotherapy, it was impossible to revise or adjust the personalized recovery plan to their new situation. The lack of this function was considered a barrier for use by those with a different course of recovery.

### Comparison With Other Studies

There are limited process evaluations concerning recovery-orientated eHealth programs available, making it difficult to compare the results with those of other similar interventions. However, 2 process evaluations performed by our research group regarding a comparable intervention have been published [21,22]. In these studies, similar positive experiences were found. The intervention was found to be supportive, participants had the feeling that they resumed activities quicker, and they found the app to be a convenient and helpful tool. The dose received and fidelity scores for the mobile app and activity tracker confirmed these findings. However, Bouwsma et al reported that some participants experienced the recovery plan being too optimistic for their own situation, whereas others found it too conservative. This is in contrast with the current findings that the recovery plan was achievable or too conservative but not too optimistic [21].

The perceived barrier of limited involvement of health care professionals is in line with findings in other studies [21]. This limited engagement was underlined by the low fidelity score of only 25% of questions asked being answered by health care professionals. A study combined Web-based modules with face-to-face coaching to guide people with early-stage dementia. Participants of this self-management program appreciated the tailored content and positive feedback of the health care professional, which increased the blended structure of the program, resulting in openness of the patients [23]. Another study provided patients a behavior change counseling monitoring and feedback tool with an activity tracker to stimulate physical activity by giving feedback on physical activity performance. This led to more discipline in carrying out activities [24]. These results are in line with our findings that participants indicated that without the activity tracker, they would have been slower in resuming activities, which is an important part of the intervention. However, participants of the program by Boots et al appreciated the personal attention given by the nurses as they combined the activity tracker with counseling in this eHealth program [23]. In both eHealth programs, participants appreciated the personal attention and the blended structure of the intervention, which was found to be lacking in the eHealth intervention in our study [23,24].

The other major barrier of our intervention was the inability to adjust or adopt the program in case of a different recovery course or additional treatment, resulting in a lack of tailored and personalized care. This is also reflected in the low fidelity score (28%) for the use of the website from those patients. In the study of Bouwsma et al, the intervention was also found to be inflexible in case of complications [21]. It is important for patients recovering after surgery to only receive information that applies to their situation, which is also confirmed in other eHealth-orientated programs [25,26]. When comparing our results with other evaluations regarding interventions for support for cancer survivors, similar results were found. Participants found the intervention to be easily accommodated into their daily routine. However, the need for more specific and tailored information was also considered important in these studies, which is comparable with our findings [27,28].

### Strengths and Limitations

A major strength of this study is the mixed-methods approach. Quality in health care is a multidimensional and complex process where some questions and information about quality of care and services are not suitable for quantification. Another strength is that the data collection and analyses were systematically performed using established theoretical frameworks for both the quantitative (the Linnan and Steckler model) and qualitative part (UTAUT framework). Usability of these frameworks was proven in previous process evaluations using these frameworks [15,29]. Another strength is that participants with both low and high intervention ratings were included in the qualitative part, resulting in both positive and critical views of the interventions being equally represented in this evaluation. A limitation of this approach is that patients who stopped prematurely with the intervention were not approached for an interview in the qualitative part because their medical health situation at that time was unknown. Reasons explaining why certain patients reported that the intervention was too difficult could provide information for the construct “effort expectancy.” This construct could be used to optimize the convenience for those patients who did not have much experience with information and communication technology or eHealth. Another limitation is the duration between using the intervention and the time the interviews were held. Time varied up to a year, potentially resulting in recall bias where some participants were not able to completely or accurately recall all the details and experiences with the “ikherstel” program. The qualitative data were only collected in a small subsample (n=14) of the study population, and therefore, these data were only presented as an example descriptively and should be interpreted with caution.

### Implications for Future Adaptations of the Intervention

To ensure a successful and sustainable implementation of the “ikherstel” program in daily practice, adaptations to the intervention are required. We suggest that health care professionals should be more involved to create more interaction. A more blended care should be introduced to solve the lack in interaction with and feedback from health care professionals. This may increase the engagement of health care professionals in the recovery process after transition to domestic recovery. We note that the hypothesis of this intervention was among others to reduce costs and time spent in the hospital by partially substituting care by this program. We must, therefore, ensure that the use of this eHealth program is additional to traditional care and prevent that care will still be duplicated. We suggest printing a recovery report that patients can take to the outpatient clinic when visiting the surgeon postoperatively to increase involvement of health care professionals. An alternative for receiving feedback would be automatically generated stimulating or confirming push messages to participants with information on their recovery progress.

The personalized recovery plan function has to be adapted so that it is more flexible and can be updated accordingly to the needs of the patient in case of an alternative disease course. The need for less generic and even more tailored information and convalescence advice was considered important by participants. In addition, the “ikherstel” program should be combined with a platform for specialized oncological aftercare for patients who will receive adjuvant chemotherapy. This enables to give patients better advice and support in the period of receiving adjuvant chemotherapy, given their treatment is not finished after the surgical procedure [30].

### Clinical Implications

This study underpins the relevance of commitment of health care professionals in supporting eHealth programs. The results reconfirmed the lack of engagement of health care professionals, despite the efforts made to improve user-friendliness and limit the time required for health care professionals. Awareness of health care professionals about eHealth possibilities has to be improved. This is important for implementing this eHealth program, because by supporting this program by health care professionals, patients who will have a colorectal resection will feel more confident and connected with the program. More involvement of health care professionals is also important for future eHealth programs where health care professionals play an important role in public engagement with eHealth and promotion of eHealth functionalities to patients in general. A possible recommendation is to include eHealth as an integral part of residents’ education and training. This could be the next step in the implementation of new technologies to the inherently changing health care system.

### Conclusions

The “ikherstel”-perioperative care program was experienced as supportive and useful in the recovery process after colorectal surgery. For this indication, the intervention needs more interaction with and feedback from health care professionals and needs more tailored guidance in case of different recovery or treatment courses. To ensure a successful implementation of the “ikherstel” program in daily practice, the awareness and involvement of health care professionals is essential. In our opinion, the recovery process of patients will benefit from an improved blended care approach that bridges the current gap between health care professionals and patients and ensures that eHealth will become part of daily practice.

## Appendix

Multimedia Appendix 1 Patient characteristics of the qualitative part of the study.

Multimedia Appendix 2 CONSORT‐EHEALTH checklist (V 1.6.1).

## Abbreviations

eConsult: electronic consult
eHealth: electronic health
RCT: randomized controlled trial
UTAUT: Unified Theory of Acceptance and Use of Technology
